# Cytomegalovirus-Associated Septic Shock Leading to Disseminated Intravascular Coagulation

**DOI:** 10.7759/cureus.82301

**Published:** 2025-04-15

**Authors:** Brandon Weissman, Sumi Singh, Taher Sbitli, Kevin Shen

**Affiliations:** 1 Otolaryngology, Lake Erie College of Osteopathic Medicine, Elmira, USA; 2 Internal Medicine, University at Buffalo, Buffalo, USA; 3 Medical School, Lake Erie College of Osteopathic Medicine, Greensburg, USA

**Keywords:** cytomegalovirus (cmv), cytomegalovirus-cmv, disseminated intravascular coagulation (dic), medical icu, severe sepsis

## Abstract

Cytomegalovirus (CMV) is typically asymptomatic in the immunocompetent but can cause deadly complications in the immunosuppressed. We report a rare case of a 58-year-old woman with stage IIIb chronic kidney disease, membranous glomerulonephritis, and diabetes mellitus who presented with profound weakness, watery diarrhea, and rectal bleeding. First managed for suspected infectious colitis, urinary tract infection, and acute kidney injury, she deteriorated quickly with confusion, hypotension, and fever. Severe metabolic acidosis, coagulopathy, and thrombocytopenia suggesting disseminated intravascular coagulation (DIC) were noted in laboratory results. The patient developed septic shock, needing intensive care, mechanical ventilation, and renal replacement therapy. CMV viral load was elevated, confirming the diagnosis of CMV-associated septic shock with DIC; the patient had an improved clinical response followed ganciclovir in conjunction with broad-spectrum antibiotics and extensive supportive care, including transfusions of fresh frozen plasma, cryoprecipitate, platelets, packed red blood cells, and vitamin K. Three weeks of targeted antiviral therapy and comprehensive supportive care allowed the patient's renal function, coagulation parameters, and clinical status to improve such that the patient could be safely discharged. This case highlights the need for early identification and very aggressive management of CMV infections in immunosuppressed patients to prevent life-threatening complications such as septic shock and DIC.

## Introduction

Cytomegalovirus (CMV) is a common virus that is exposed to approximately 59% of the population older than six years of age. It is a double-stranded DNA virus that is a part of the herpes virus family [1]. Transmission can occur in many ways, such as through blood/organ transplantation, breastfeeding, perinatally, and bodily fluids [2]. Infection with CMV in healthy adults is most commonly asymptomatic [3]. Immunocompromised patients can have an asymptomatic course; however, oftentimes, these patients develop viremia with episodes of fevers and potential involvement of the liver, lungs, gastrointestinal tract, or retinal involvement [4]. This can manifest as hepatitis, pneumonia, colitis, and retinitis. Laboratory viral identification is the preferred initial test for patients with suspected CMV infection [1]. Immunocompetent infections do not require treatment; however, for immunocompromised patients, antivirals such as cidofovir, foscarnet, ganciclovir, and valganciclovir are used [1]. CMV is rarely associated with septic shock and disseminated intravascular coagulation, with few past reports highlighting this. We present a case of a woman who presented with confusion and ultimately developed sepsis and disseminated intravascular coagulation (DIC) from CMV.

## Case presentation

A 58-year-old woman presented to the emergency department with significant weakness and the inability to take care of herself. She had a past medical history of stage IIIb chronic kidney disease, nephrotic syndrome, anemia, type 2 diabetes mellitus, diabetic nephropathy, membranous glomerulonephritis, hypertension, hypothyroidism, and hypercholesterolemia. The patient was also treated for a urinary tract infection (UTI) with nitrofurantoin 10 days before presentation.

Upon questioning, the patient stated that since the previous day, she could barely walk and was so weak she could not lift herself off the bed. She also noted significant watery diarrhea and rectal bleeding for a one-week duration. The patient endorsed feeling cold but denied fever, chest pain, shortness of breath, or abdominal pain. She also stated that the UTI symptoms had resolved. The laboratory values on arrival are listed in Table 1.

**Table 1:** Laboratory values on initial presentation K/uL: thousands per microliter, g/dL: grams per deciliter, fL: femtoliters, mg/dL: milligrams per deciliter, mmol/L: millimoles per liter, mmHg: millimeters of mercury, mEq/L: milliequivalents per liter, PCO2: partial pressure of carbon dioxide, PO2 partial pressure of oxygen, HCO3: bicarbonate

Parameter	Patient results	Reference range
White Blood Cells	7.6 K/uL	4.5-11.0 K/uL
Hemoglobin	9.4 g/dL	12.0-16.0 g/dL
Hematocrit	28.6	36.0-47.0%
Mean Corpuscular Volume	87.1 fL	78.0-100.0 fL
Platelet Count	203 K/µL	145-450 K/µL
Glucose	152 mg/dL	74-99 mg/dL
Calcium	7.3 mg/dL	8.4-10.2 mg/dL
Sodium	128 mmol/L	133-145 mmol/L
Potassium	5.4 mmol/L	3.3-5.1 mmol/L
Potential of hydrogen	7.14	7.35-7.45
PCO2 Venous	32 mmHg	40-60 mmHg
PO2 Venous	55 mmHg	30-50.0 mmHg
HCO3	10.9 mEq/L	22-28 mEq/L
Creatinine	4.16 mg/dL	0.6-1.2 mg/dL

**Figure 1:**
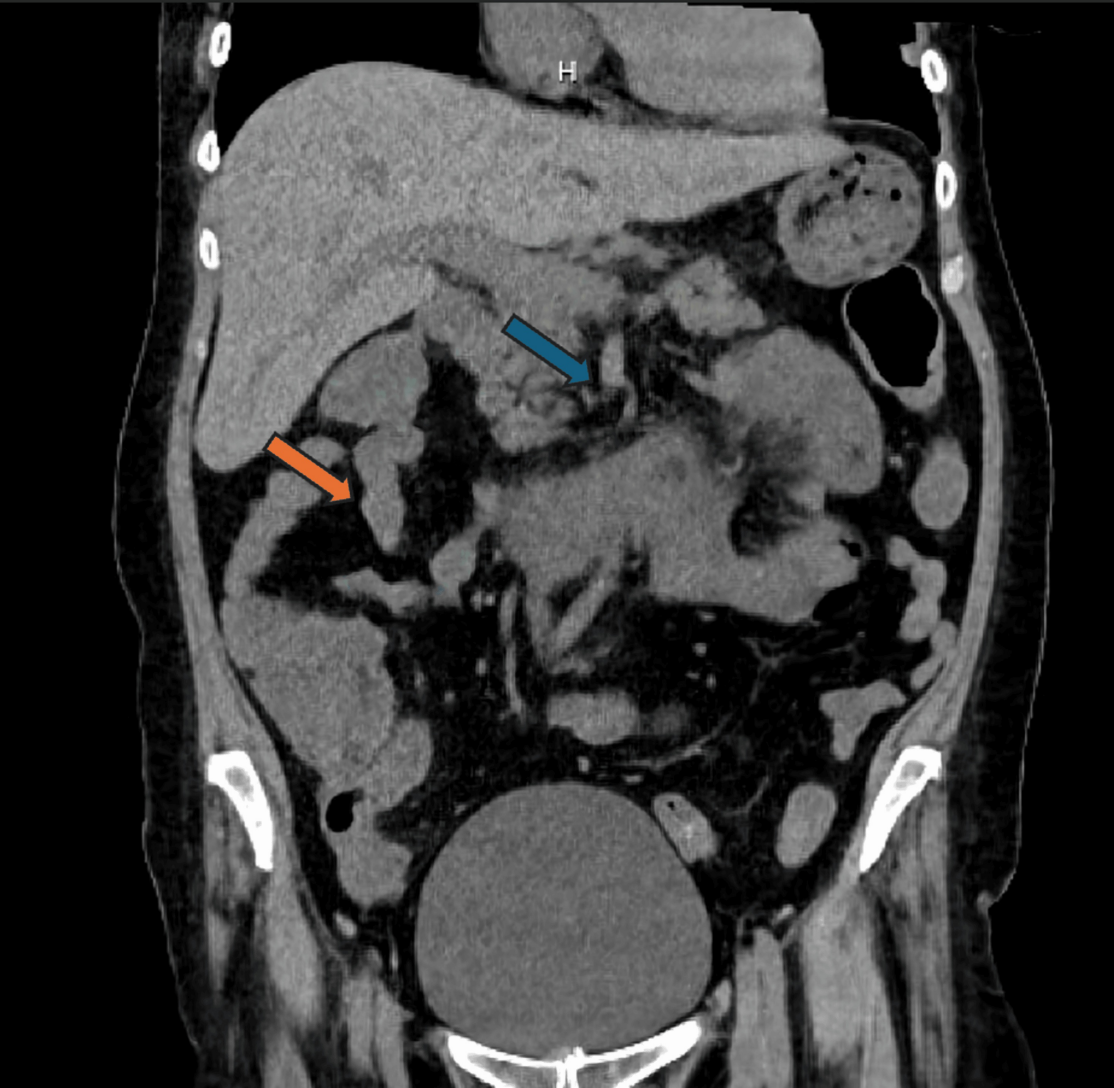
Coronal computed tomography scan of the abdomen; orange arrow represents the descending colon and blue arrow represents the transverse colon

The patient was admitted to the medical service. On admission, a computed tomography (CT) scan was completed and showed a nonspecific abdomen with no signs of obstruction or free air, with an element of ascending and transverse colon thickening suggestive of inflammatory colitis, as seen in Figure 1. The patient was treated for acute kidney injury superimposed on chronic kidney disease stage IIIb. She was placed on an intravenous drip with bicarbonate for the diarrhea due to suspected colitis. She was treated with intravenous ceftriaxone (Rocephin) and metronidazole (Flagyl) for a possible UTI. The patient’s anemia was thought to be caused by chronic kidney failure, and she was transfused with one unit of packed red blood cells. 

One week after admission, the patient’s blood pressure was 72/47 mmHg, heart rate was 91 beats per minute, temperature was 101^o^F, and the patient was not oriented to herself, time, or place and was transferred to the intensive care unit (ICU). The international normalized ratio (INR) was 3.5, with an elevated D-dimer, bilateral lower extremities were positive for deep venous thrombosis, platelets count progressively declined, and the patient was diagnosed with disseminated intravascular coagulation. The laboratory values are seen in Table 2.

**Table 2:** Laboratory values on initial intensive care unit transfer mg/dL: milligrams per deciliter, U/L: units per liter, mmol/L: millimoles per liter, K/uL: thousands per microliter, g/dL: grams per deciliter, ng/ml: nanograms per milliliter, INR: international normalized ratio

Parameter	Patient Results	Reference Range
Prothrombin Time	42.3 seconds	9.3-13.5 seconds
Activated Partial Thromboplastin Time	105 seconds	25-35 seconds
Fibrinogen	123 mg/dL	200-400 mg/dL
INR	3.5	0.9-1.2
Lactate Dehydrogenase	2152 U/L	105-210 U/L
Lactic Acid	6.2 mmol/L	0.5-2.0 mmol/L
Red Blood Cell Count	2.57 K/uL	4.10-5.60 K/uL
White Blood Cells	8.8 K/uL	4.5-11.0 K/uL
Hemoglobin	7.4 g/dL	12.0-16.0 g/dL
Hematocrit	23.1%	36.0-47.0%
Platelet Count	54 K/µL	145-450 K/µL
D-dimer	4,199 ng/ml	<=500 ng/ml

**Figure 2:**
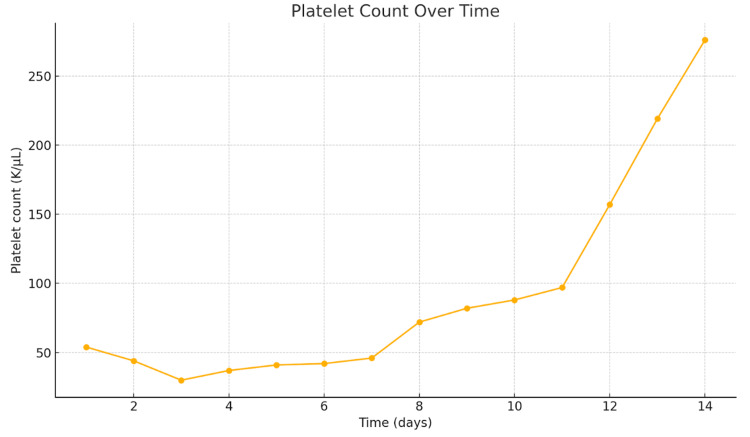
Platelet count from initial diagnosis of disseminated intravascular coagulation Platelet count is crucial in disseminated intravascular coagulation (DIC) because widespread clot formation consumes platelets, leading to thrombocytopenia, which increases the risk of bleeding. A rapidly decreasing platelet count is a key marker of ongoing coagulation dysfunction and helps differentiate DIC from other causes of coagulopathy.

**Figure 3:**
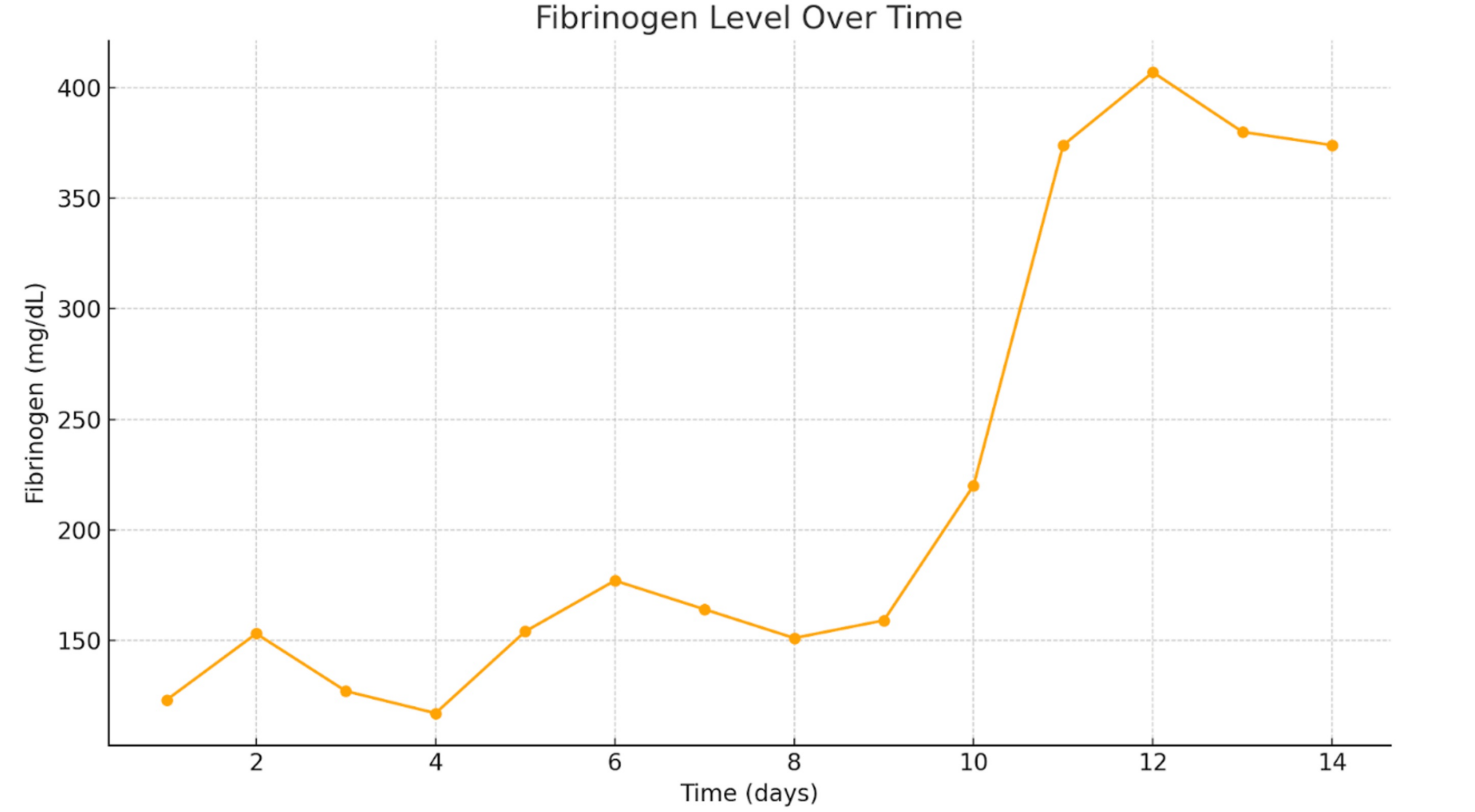
Fibrinogen level from initial diagnosis of disseminated intravascular coagulation Fibrinogen is a glycoprotein synthesized by the liver that plays a crucial role in blood clot formation by converting into fibrin during coagulation. In disseminated intravascular coagulation (DIC), fibrinogen levels decrease due to excessive clotting and fibrinolysis, contributing to both thrombosis and bleeding as fibrinogen is rapidly consumed and degraded.

The patient was treated with 5 milligrams (mg) of vitamin K for three days and a heparin drip at 40 units/kg every six hours. The patient also received one unit of packed red blood cells (PRBC), cryoprecipitate, fresh frozen plasma (FFP), and platelets. The patient's hypotension was not responsive to fluids and required a norepinephrine infusion for pressure support. Methicillin-resistant-*Staphylococcus aureus* swabs of the nose were taken and were negative. Multiple respiratory and blood cultures were negative. CMV and Epstein-Barr virus (EBV) tests were conducted per our infectious disease team. Serum CMV DNA QN real-time polymerase chain reaction (PCR) came back elevated at 27,443 IU/mL; the patient was promptly started on meropenem 1 g and ganciclovir 107.5 mg for the CMV viremia and possible another source of infection, although none was found. She was intubated during the hospitalization due to acute hypoxic respiratory failure and was maintained on dialysis. The patient was treated with ganciclovir 107.5 mg for three weeks, and her blood count improved, labs improved, and kidney function improved, and she was discharged. The improvement in fibronogen level and platelet count is seen in Figure 2 and Figure 3.

## Discussion

This patient initially presented with significant watery diarrhea and rectal bleeding and was initially admitted to our medical service, which focused on antibacterial therapy and treatment for her acute kidney injury, given her elevation in baseline creatine and possible UTI. However, the hospital course was complicated by a period of significant confusion, hypotension, and fever, which required the patient to be transferred to the ICU. The patient’s laboratory findings were positive for significantly elevated prothrombin time (42.3 seconds), activated partial thromboplastin time (105 seconds), low fibrinogen (123 mg/dL), elevated D-dimer (4,199 ng/mL), and thrombocytopenia (platelet count 54 K/µL) which were consistent with DIC, indicating consumption of platelets and coagulation factors. The patient also became increasingly hypotensive, tachycardic at times, and hypoperfused (evidenced by elevated lactate), fulfilling clinical criteria for septic shock. Management of DIC was focused on replacing depleted coagulation factors and cellular components through transfusions of fresh frozen plasma, cryoprecipitate (for fibrinogen), platelet concentrates, and PRBC. Vitamin K therapy was administered to ensure adequate hepatic production of vitamin K-dependent clotting factors (II, VII, IX, and X) [5]. These combined interventions stabilized the patient’s hemodynamics, improved coagulation parameters, and supported end-organ function. 

The patient’s diarrhea had not improved, and multiple *Clostridium difficile* tests were conducted, which were negative. Our infectious disease service recommended EBV and CMV testing, which resulted in a positive CMV test. The serum CMV result was significant for CMV infection. The patient’s immunosuppression increased her susceptibility to CMV. Reactivation of a dormant infection is the most common cause in immunosuppressed patients. In these patients, CMV can invade various organ systems, causing organ failure and, ultimately, sepsis and mechanical ventilation [6]. In the present case, CMV appeared to precipitate a septic picture. Although CMV is less commonly associated with fulminant septic shock, it can nonetheless trigger a robust inflammatory response characterized by excessive cytokine release. 

This response, alongside the patient’s comorbidities, likely contributed to endothelial injury, hypercoagulability, and, ultimately, DIC. CMV in vitro studies have shown that CMV-infected endothelial cells can develop a procoagulant response [7]. Past literature has reported CMV-related DIC [8]. Therefore, this patient's DIC can be attributed to CMV.

Therapy focused on eradicating the underlying cause of infection while providing supportive care for DIC. Ganciclovir was initiated due to its proven efficacy against CMV. It works by inhibition of replication by potent inhibition of viral DNA polymerase [9]. Ultimately, this timely and targeted approach allowed the patient’s blood counts, renal status, and coagulation profile to recover, facilitating her discharge after three weeks of antiviral therapy.

## Conclusions

This case shows how a patient who is immunocompromised and has multiple comorbidities can have life-threatening complications from CMV. When CMV proliferates rapidly, it may induce a severe inflammatory response that can include septic shock and possibly DIC, as has been seen in this report. This type of management was successful in this particular case in that it was based on antiviral therapy with ganciclovir to inhibit viral replication, broad-spectrum antibiotics for possible bacterial co-infection, and other means of managing the coagulopathy and organ dysfunction. The underlying infection was treated, and aggressive supportive care was provided to enhance the patient’s coagulation function, renal function, and general condition so that the patient could be discharged safely. This case report highlights the rarity of CMV-associated DIC and is an important differential that clinicians must recognize. 
